# Comparison of Effects and Brain-Gut Regulatory Mechanisms of Acupuncture and Flunarizine for Migraine: Study Protocol for a Randomized Controlled Trial

**DOI:** 10.1155/2021/5676718

**Published:** 2021-01-07

**Authors:** Ya-Nan Wang, Ming-Sheng Sun, Xi-Xiu Ni, Tian Tian, Lu Liu, Xiao Li, Tao Xu, Si-Yuan Zhou, Jiao Chen, Fan-Rong Liang, Ji-Xin Liu, Ling Zhao

**Affiliations:** ^1^Acupuncture and Tuina School, Chengdu University of Traditional Chinese Medicine, No. 37 Shier Qiao Road, Chengdu, Sichuan 610075, China; ^2^Center for Brain Imaging, School of Life Science and Technology, Xidian University, No. 266 Xinglong Section, Xifeng Road, Xi'an, Shaanxi 710126, China

## Abstract

**Background:**

As a central nervous system disease, migraine often coexists with gastrointestinal disorders, which suggests a disruption of brain-gut regulation. Clinical studies have confirmed that acupuncture and flunarizine not only alleviate migraine attacks but also substantially inhibit accompanying gastrointestinal symptoms. However, it is still not clear how acupuncture and flunarizine regulate the interactions of brain, gut, and microbiome. Therefore, this study will combine neuroimaging technology and gut microbiota detection technology to explore and compare the effects and brain-gut modulating mechanisms of acupuncture and flunarizine for migraine.

**Methods:**

This randomized clinical trial will recruit 66 patients with migraine without aura. Participants will be randomly assigned in a 1 : 1 ratio to an acupuncture group or a control group. The acupuncture treatment strategy is based on experience from our previous study and consensus meetings with clinical experts. Patients will receive 12 sessions of manual acupuncture treatment (once every other day to a total of three times per week, followed by a 2-day break). Flunarizine will be administered at a dose of 5 mg daily in the control group. Participants in both groups will receive treatment for a period of 4 weeks. The primary outcome is the change in frequency of migraine attacks, and the secondary outcomes include the changes in migraine days (days on which migraine attacks occurred), average migraine severity, gastrointestinal symptoms, psychiatric symptoms, and quality of life. Fresh stool samples will be collected, and 16S ribosomal RNA gene sequencing analysis will be used for gut microbiota. Magnetic resonance imaging will be applied to detect between-group changes in brain function. The abovementioned indicators will be collected at baseline, after a 4-week intervention, and at the 12-week follow-up. *Discussions.* From the perspective of brain-gut regulatory mechanisms, we will combine brain neuroimaging and gut microbiological data to partially reveal the similarities and differences of acupuncture and flunarizine on the treatment of migraine. The trial is registered with ChiCTR2000034417.

## 1. Introduction

Migraine is a neurological disorder characterized by recurrent attacks, high clinical incidence, and obvious disability. A latest systematic analysis of the Global Burden of Disease study reported that 1.25 billion people suffered from migraine in 195 countries and territories [1]. Migraine ranked second globally in terms of years lived with disability [2] and was the leading cause of disability for individuals at the age of younger than 50 years [3]. Migraine increases the risk of cardiovascular and cerebrovascular diseases [4–6] and may even lead to self-harm and suicide in severe attacks [7], which brings extensive negative impacts to patients' physical and mental health.

Previous studies have found that migraine attacks can be triggered by foods such as alcohol, chocolate, and cheese [8], and the number of headache days and episodes are greatly reduced during fasting periods [9], indicating that migraine is associated with the disruption of brain-gut interactions [10]. Migraine often coexists with gastrointestinal disorders [11, 12], commonly accompanied by nausea, vomiting, and other gastrointestinal symptoms [13, 14], which suggests that the gut microbiota may play a key role in migraine via brain-gut interactions [15]. Migraine attacks can alter gut microbiota composition [16], while gut microbiota may be related to migraine pathogenesis [17]. Several studies have shown that probiotics are effective in reducing the intensity and frequency of migraine attacks and may substantially reduce the consumption of medication and the incidence of migraine disability [18, 19]. Moreover, the traditional Chinese herb *Gastrodia elata*, which is often used to treat headache or migraine, has been reported to promote migraine relief and modulate gut microbiota [20]. A report released by the Rome Working Team has confirmed that brain-gut interactions are associated with changes in the neural, endocrine, and immune pathways [21]. On the basis of the abovementioned evidence, we suggest that there is an interaction between the brain and the gut in migraineurs.

Pharmacological and nonpharmacological therapies have been used to prevent migraine attacks. The calcium channel blocker flunarizine, which is recommended as a first-line prophylactic medication in several guidelines [22–25], has been shown to be effective in relieving gastrointestinal manifestations in addition to reducing the frequency and duration of migraine attacks [26–28]. In terms of nonpharmacological therapy, acupuncture has been widely recognized as an effective treatment for migraine prophylaxis [29]. As acupuncture has beneficial effects and mild side effects [30], migraineurs have expressed good compliance and high satisfaction with acupuncture therapy [31, 32]. Our previous studies have confirmed that acupuncture treatment has a good effect on relieving migraine attacks and digestive symptoms [33, 34]. There is evidence that acupuncture can regulate gut microbiota disorders and improve the diversity and richness of bacteria species in the gut [35, 36]. Several lines of research on the use of acupuncture and flunarizine for migraine have shown that flunarizine has a good effect in reducing pain intensity and improving quality of life, and in addition to the effects mentioned above, acupuncture can effectively reduce the number of migraine days and regulate psychological state [37, 38].

Previous studies have revealed substantial abnormalities in brain functional activities and in the species diversity and metabolic functions of gut microbiota in migraine patients compared with healthy controls [15, 39]. However, there is no report on whether clinical symptoms are relieved by medication and acupuncture is related to the regulation of brain-gut-microbiota interactions in migraineurs to date. Therefore, using neuroimaging and gut microbiota examination, we aim to examine the effect of acupuncture and flunarizine treatment for migraine and compare the similarities and differences in gut microbiota and brain functional network elicited by acupuncture and flunarizine treatment. Furthermore, a tripartite network analysis based on graph theory will be used to explore the relationships among brain network activities, gut microbiota, and clinical efficacy under the acupuncture and flunarizine for migraineurs.

## 2. Methods and Design

### 2.1. Objectives

This study aims to (1) re-evaluate the efficacy of acupuncture and flunarizine on migraine treatment; (2) compare the similarities and differences in brain activity and gut microbiota associated with acupuncture and flunarizine treatment for migraine; and (3) investigate the correlation between symptom improvement, brain activity changes, and gut microbiota regulation elicited by acupuncture and flunarizine.

### 2.2. Trial Design

**Figure 1:**
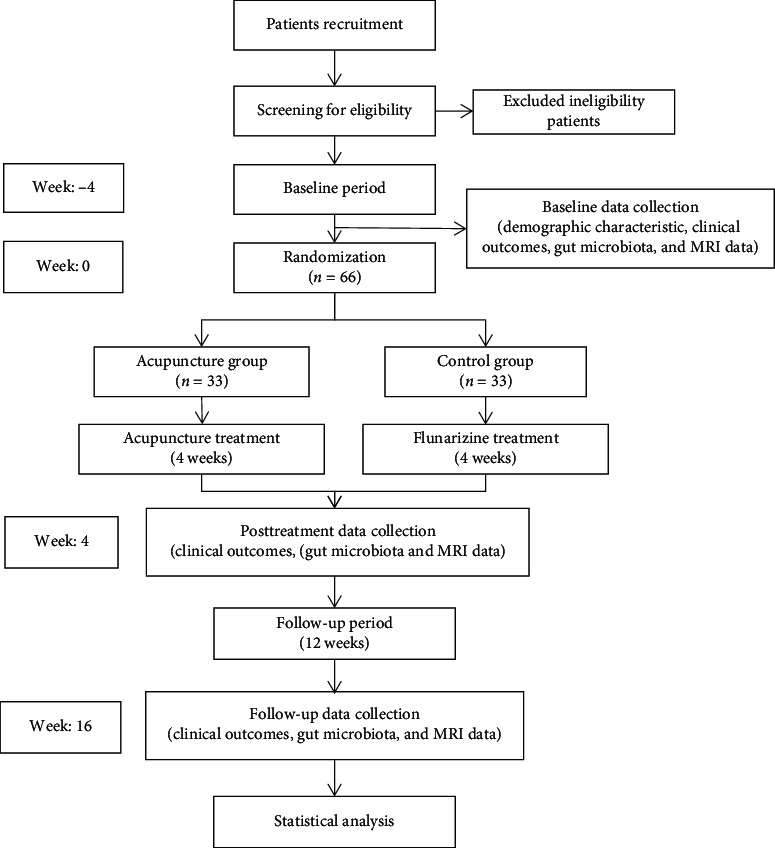
Flowchart of the trial design.

Study participants will be patients who have migraine without aura (MwoA). A total of 66 patients meeting the diagnostic criteria of the International Classification of Headache Disorders [40] will be recruited and randomly assigned to two groups (1 : 1 allocation ratio) to receive either acupuncture or drug therapy. This study protocol was developed in accordance with the Standard Protocol Items: Recommendations for Intervention Trials (SPIRIT) [41]. This trial has been registered with the Chinese Clinical Trial Registry (ChiCTR). The flowchart of the trial design is shown in Figure 1.

### 2.3. Participants

All potential MwoA patients meeting the diagnostic criteria will be recruited from the outpatient department of neurology at the Hospital of Chengdu University of Traditional Chinese Medicine (TCM). A recruitment advertisement for this trial will also be posted on the WeChat platform. Only MwoA patients who are completely in conformity with the inclusion criteria will be recruited to the trial.

#### 2.3.1. Inclusion Criteria

Eligible participants must meet the following criteria: (1) men or women aged 18–55 years; (2) right handed, length of education ≥6 years; (3) a history of migraine of, at least, 1 year with the first onset before the age of 50 years; (4) migraine attacks ≥2 times and number of migraine days <15 days per month during the past 3 months and baseline observation; (5) completion of the baseline headache diary; and (6) provision of written informed consent.

#### 2.3.2. Exclusion Criteria

Patients matching any of the following criteria will be excluded: (1) any combination of head trauma history, secondary headache, tension-type headache, intermittent-type headache, cluster-type headache, or cardiovascular and digestive diseases; (2) a history of major intestinal resection or gastrointestinal surgery; (3) presence of psychiatric disease, infection, bleeding disorders, or allergic constitution; (4) pregnancy, lactation, or plans to become pregnant within 6 months; (5) inability to understand or fill in a headache diary; (6) nuclear magnetic contraindications, such as claustrophobia; (7) a history of taking antibiotics or receiving acupuncture therapy for 3 days or more in the past 3 months; (8) long-term use of analgesic drugs and medications to prevent migraine attacks; and (9) participation in a similar study in the last 3 months.

### 2.4. Data Collection

At the 4-week baseline, patient demographic surveys (age, sex, height, weight, education, disease course, and medical history) will be administered. Clinical outcomes, fecal samples, and magnetic resonance imaging (MRI) data will be collected at the 4-week baseline, end of the 4-week intervention, and at the 12-week follow-up (Table 1).

#### 2.4.1. Clinical Data Collection

All participants will be instructed to complete a headache diary every 4 weeks during the study. The headache diary will mainly record the time of migraine onset, migraine duration, migraine severity (detected by a visual analog scale), and use of rescue medicine.

The following clinical outcomes will be assessed by independent assessors who have been trained before participating in the study. The primary outcome is the change in frequency of migraine attacks, and the secondary outcomes include the changes in migraine days (days on which migraine attacks occurred), average migraine severity, gastrointestinal symptoms, psychiatric symptoms, and quality of life. The intensity and frequency of gastrointestinal symptoms will be assessed using the Gastrointestinal Symptom Rating Scale (GSRS) [42, 43]. As migraine is often accompanied by psychiatric symptoms, the Beck Anxiety Inventory (BAI) [44] and the Beck Depression Inventory (BDI) [45] will be used to measure the emotional states of migraineurs, and quality of life will be assessed using the Migraine-Specific Quality of Life Questionnaire (MSQ) [46].

#### 2.4.2. Gut Microbiota Collection

Fecal samples will be collected from 66 MwoA patients at baseline, end of the 4-week intervention, and at the 12-week follow-up. Patients will be instructed to maintain a regular diet and refrain from spicy, oily food, and any food containing probiotics (e.g., yogurt) for 1 week before the fecal sample collection. Migraineurs will receive a teaching video, as well as detailed written and oral instructions, on how to collect feces using a Longseegen stool storage kit (Longsee Medical Technology Co. LTD., Guangdong, China) in the morning after an overnight fast. The collection time of the fecal sample will be ±1 days for MRI examination, and the collection sites can be selected at home or in the hospital according to patients' preference. The inside of the middle section of the feces (approximately 3 g) will be collected and then immediately frozen at −80°C in the hospital or briefly stored in a domestic refrigerator at 4°C before being transferred to researchers within 24 hours for subsequent analysis.

#### 2.4.3. MRI Data Acquisition

An MRI scan will be carried out using a 3.0TGE Signa MR750 system (GE Healthcare, Milwaukee, USA) in the Hospital of Chengdu University of TCM. High-resolution 3D T1-weighted anatomical images using a 3D spoiled gradient-recalled sequence will be produced. The acquisition parameters are as follows: repetition time (TR)/echo time (TE) = 5.97/1.96 ms, field of view (FOV) = 240 × 240 mm^2^, flip angle = 12°, matrix size = 512 × 512, 156 slices, and voxel size = 1 × 1 × 1 mm^3^.

Resting-state fMRI images will be obtained using a gradient-recalled echo planar imaging sequence with the following parameters: TR/TE = 2000/30 ms, FOV = 220 × 220 mm^2^, flip angle = 90°, matrix size = 64 × 64, 43 transverse slices without slice gap, voxel size = 3.75 × 3.75 × 3.2 mm^3^, and a total of 266 volumes for each subject.

MRI will be performed during the periovulatory phase (days 12–16 of the menstrual cycle) [47, 48]. All participants will be free from a typical migraine attack for, at least, 1 week prior to the MRI scan [47]. During the examination, subjects will be instructed to lie down with their eyes closed, not to think of anything, and particularly not to fall asleep. Foam padding will be used to restrict head motion, and earplugs will be used to attenuate scanner noise.

### 2.5. Sample Size, Randomization, and Blinding

#### 2.5.1. Sample Size Calculation

Following a previous study, we anticipate that the change in the average number of migraine days in the acupuncture group will be 4.1 ± 3.5, and in the flunarizine group, it will be 1.9 ± 2.3 [37]. Assuming that *α* = 0.05 and 1 − *β* = 0.80, 30 patients will be required for each group, as calculated using *G*^*∗*^Power V3.1.9.2 software. There is no consensus on sample size for neuroimaging and gut microbiota research. A review of previous similar studies suggests that 12–27 individuals in each group is a reasonable sample size for stable and reliable results [49, 50]. Finally, a total of 66 patients will be recruited to compensate for a withdrawal rate of 10%.

#### 2.5.2. Randomization

To avoid possible selective bias, a statistician will use Statistical Product Service Solutions (SPSS) Statistics V25.0 to generate a table of random numbers that will be placed in opaque envelopes. This randomization scheme will be kept secure by a specially assigned person. The researchers will contact this designated person by telephone to acquire the corresponding random number and grouping for each participant enrolled in the study.

#### 2.5.3. Blinding

Owing to the difference between the two therapies, double-blind will be difficult to implement in this study. However, the purpose of blinding is to avoid the influence of subjective factors from the subjects and researchers on the study results. In order to minimize the subjective influence, we will ensure that evaluation and statistical analysis are carried out by a third party who is unaware of the grouping.

### 2.6. Intervention

The interventions will be implemented strictly in accordance with the Consolidated Standards of Reporting Trials (CONSORT) [51] and the Standards for Reporting Interventions in Clinical Trials of Acupuncture (STRICTA) [52].

#### 2.6.1. Acupuncture Group

**Figure 2:**
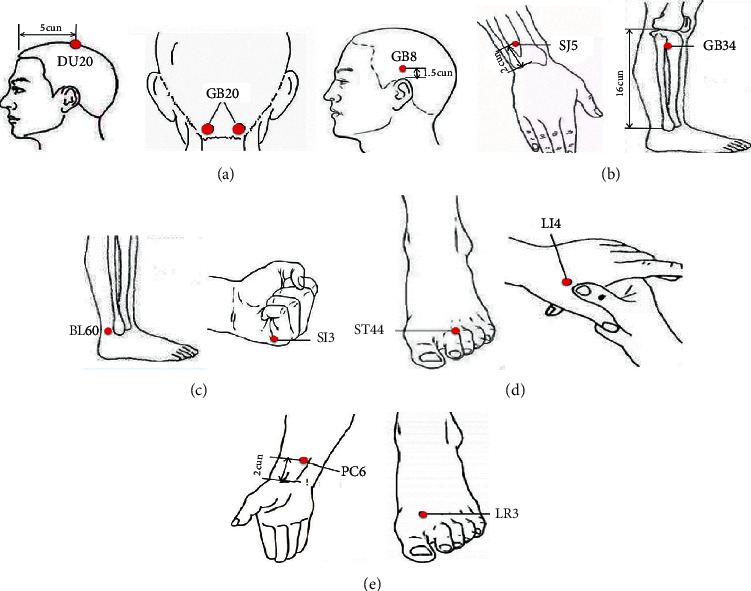
Acupoints of acupuncture treatment used in this study. (a) Main prescription; (b) Shaoyang headache; (c) Taiyang headache; (d) Yangming headache; and (e) Jueyin headache.

In the acupuncture group, the needles will be placed at obligatory acupoints Fengchi (GB20), Shuaigu (GB8), and Baihui (DU20) in all treatments. Using syndrome differentiation, acupoints from other meridians will be chosen according to the location of headache as follows: Shaoyang headache: Waiguan (SJ5) and Yanglingquan (GB34); Taiyang headache: Kunlun (BL60) and Houxi (SI3); Yangming headache: Hegu (LI4) and Neiting (ST44); and Jueyin headache: Taichong (LR3) and Neiguan (PC6) (Table 2 and Figure 2). Except DU20, other acupoints will be selected bilaterally in this prescription. Acupoints other than the prescribed ones will not be used. The treatment strategy is based on experience from our previous study and consensus meetings with clinical experts [33]. Disposable sterile acupuncture needles (25 to 40 mm in length and 0.25 mm in diameter) will be used in the treatment. Each point will be needled to achieve the Deqi sensation, and needles will be retained for 30 minutes per session. Patients will receive 12 sessions of manual acupuncture treatment (once every other day to a total of three times per week, followed by a 2-day break) for a period of 4 weeks. Treatments will be performed by licensed acupuncturists who have received more than 3 years of training.

#### 2.6.2. Control Group

In the control group, flunarizine (manufacturer: Chongqing Kerui Pharmaceutical Co., LTD., Chongqing, China; SFDA approval number: H19994054) will be administered at a dose of 5 mg daily, following migraine guidelines and the professional advice of neurologists [22–25]. Patients will take the capsules continuously for 4 weeks without interruption.

In principle, participants should not take any analgesic medication during the study period. However, ibuprofen sustained-release capsule (Manufacturer: Tianjin Smith Kline & French laboratories, LTD., Tianjin, China; SFDA approval number: H20013062) can be taken as a rescue medicine for unbearable headaches, but the usage must be recorded in the headache diary.

### 2.7. Data Management and Quality Control

To ensure successful study implementation, all researchers will receive standardized training before the research starts. Clinical data will be recorded and managed by two trained graduate students using paper and electronic case report forms (CRFs). To avoid data entry errors, data will be proofread by two independent researchers when entering the electronic CRFs. All study data will be entrusted to the Brightech-Magnasoft Clinical Information Management System (CIMS) (address: 285 Davidson Avenue, Suite 504, Somerset, NJ 08873, phone: 908-790-8888, website: http://www.brightech-intl.com) for data management and analysis. MRI and gut microbiota data will be collected strictly in accordance with the operating procedures and trial design.

### 2.8. Safety and Monitoring

During the study, patients receiving acupuncture or flunarizine treatment may experience adverse events, such as pain, fainting, bleeding, hematoma, infection (acupuncture treatment), drowsiness, and fatigue (flunarizine treatment). Regardless of whether the adverse events are related to the treatment in this study, they will be recorded in detail and handled properly. Serious adverse events will be immediately reported to the primary investigator to assess whether to proceed with the study.

### 2.9. Statistical Analysis

#### 2.9.1. Clinical Data Analysis

The analysis plan will be determined before the study is conducted. Demographic characteristics, baseline characteristics, and clinical outcomes will be analyzed using SPSS25.0 statistical software based on the intention-to-treat population, which comprises subjects who receive, at least, one treatment and undergo, at least, one posttreatment efficacy evaluation. Missing data will be replaced by data from the last observation. Normally distributed continuous variables will be presented by means and standard deviations with 95% confidence intervals. Nonnormally distributed continuous variables will be described using medians and interquartile ranges. Categorical variables will be described using percentages and frequencies. The significance level used for statistical analysis with 2-tailed testing will be 5%. Qualitative data will be compared using the chi-square test. If the quantitative data are normally distributed, we will use analysis of variance to detect differences between the two groups; if not, the Mann–Whitney test will be used. Data sheets will be anonymized for data entry, and statistical analysis will be conducted by an independent statistician blinded to treatment allocation.

#### 2.9.2. Gut Microbiota Analysis

High-throughput sequencing will be used to detect gut microbiota information. Sequencing will be performed on the V4 region and the V3 to V4 amplification region of the 16S ribosomal RNA (rRNA) gene. The PE300 sequencing strategy will be used to carry out high-throughput sequencing on the Illunima Miseq platform (Illumina, San Diego, USA). The results will be analyzed using biological information after sequencing, and Pearson correlation analysis will be used to examine correlations between different indicators. QIIME software (http://qiime.org) will be used for operational taxonomic unit (OTU) clustering. The OTU representation sequence will be calculated using the Ribosomal Database Project Classifier based on the naïve Bayesian principle and compared with the reference sequences in the Silver database to obtain the species classification information corresponding to the OTU. The OTU cluster analysis results will be used to calculate the abundance index (Abundance-based Coverage estimator and Chao1 estimator), diversity index (Simpson and Shannon), and uniformity index to evaluate alpha diversity. Principal component analysis and principal coordinate analysis will be used to characterize beta diversity in the different groups.

#### 2.9.3. MRI Data Analysis

Brain parcellation will be performed using FreeSurfer (Dale, Fischl et al.1999; Fischl, Sereno et al. 1999; Fischl, Salat et al. 2002), and then, the parcellation and the functional connectivity will be combined to construct a functional brain network. FMRIB Software Library (FSL) developed by Oxford University (https://fsl.fmrib.ox.ac.uk/fsl/fslwiki/FSL) will be used to process high-resolution 3D T1-weighted anatomical images. The resting-state images will be preprocessed using the MATLAB toolkit SPM8 (http://www.fil.ion.ucl.ac.uk/spm/software/spm8) and resting-state fMRI (DPARSF) data processing kit (Resting-State fMRI Data Analysis Toolkit, REST, http://www.restfmri.net). Region-to-region functional connectivity will be computed between all the brain regions in CONN toolbox version 13 (http://www.nitrc.org/projects/conn). The connectivity correlation coefficients will then be used to construct the final functional network. Finally, the Graph Theoretic GLM tool (http://www.nitrc.org/projects/metalab_gtg) and in-house MATLAB workflow scripts will be used to calculate and analyze the functional brain network properties and organization from the subject-specific functional brain networks, and then, regional weighted network metrics indexing centrality will be computed.

#### 2.9.4. Tripartite Network Analysis

A tripartite network analysis based on graph theory will be used to compare the interactions among brain network activity, gut microbiota, and clinical data for migraineurs in the acupuncture and flunarizine groups. The interactions between the phenome (clinical symptoms), gut microbiome (fecal microbial community), and connectome (indices of regional centrality in the brain) will be analyzed using Spearman's correlations in MATLAB R2015b. Fisher's *r*-to-*z* transformation will be used to measure the correlation coefficient difference between the acupuncture and flunarizine group. The *Z* test will be applied, and an uncorrected value of *p* < 0.05 will be considered statistically significant. The software Cytoscape 3.7.2 (https://cytoscape.org) will be used to visualize and generate a brain activity, gut microbiota community, and clinical data interaction network. This association of this network will be included if Pearson's correlation for the data of the different groups achieves significance. The architecture of different group networks will be based on a significantly different network. The results will be described in terms of direct (correlations with microbial community) and indirect effects (clinical symptoms and brain connectivity metric) with an emphasis on the interaction between microbiome and clinical symptoms. An independent sample *t*-test will be used to examine between-group differences in clinical variables.

## 3. Discussion

The pathogenesis of migraine is still controversial, the main reason of which is that multiple factors are involved, such as blood vessels, neurons, inflammatory mediators, abnormal brain structure and function, and genetic inheritance [53]. However, the recent research related to the regulation mechanism of brain-gut interaction seems to shed new light on the pathogenesis of migraine [17]. The brain-gut interaction is considered an important regulatory mechanism to integrate the brain with the gastrointestinal tract. Research on an integrative model for gut-brain interaction disorders has confirmed that the mediation in gut-to-brain communication and brain-to-gut communication is performed by different pathways [21]. However, information transmission between the gastrointestinal tract and the brain is mainly conducted via the nerve, endocrine, immunity, and gut microbiota pathways [54]. Gut microbiota is closely related to human health and disease through a long-term evolutionary selection process [55]. Gut microbiota and its metabolites are involved in the regulation of brain function and behavior [56, 57]. A lack or deficiency of gut microbiota can affect the normal development of the central nervous system [58], and such abnormality obviously exists in the gut microbiota structure of patients with central nervous system disorders [59–61].

Multiple factors are involved in the interrelationship of the brain and gut in migraine, such as neuropeptides, inflammatory mediators, gut microbiota, stress hormones, and nutritional substances [16]. A latest study has found the differences in composition and function of gut microbiota in the comparison of migraineurs and healthy control [15]. Moreover, previous fMRI studies have confirmed that abnormal architecture and connectivity in the brain network of migraineurs [62–64]. Recently, a human research has identified that the shifts of gut microbiota composition profile are correlated with the signal changes of brain activities involved in the cingulum and cerebellum [65].

Several studies linked to the effects of acupuncture on the structural characteristics of gut microbiota have shown that acupuncture stimulation can substantially improve the diversity of gut microbiota and the content of beneficial microflora [36, 66, 67]. Our previous studies have indicated that the analgesic effect of acupuncture may be related to the findings that acupuncture could regulate the functional connectivity of brain and the response of brain regions in the pain matrix in migraineurs [39, 68]. It seems to be a new direction and strategy to explore the brain-gut regulatory mechanisms of migraine treatment in the combination of neuroimaging and gut microbiological analysis. fMRI is a dynamic visualization technology to observe brain activities on the basis of different blood-oxygen state in various brain regions and is extensively applied in the acupuncture mechanism for pain diseases [69, 70]. The gut microbiological analysis is based on 16S rRNA high-throughput sequencing of fecal samples, which can amplify 16S rRNA fragments using a polymerase chain reaction to achieve taxonomic identification and accurate quantification of the gut microbiota community. At present, this technology has been widely used in the diagnosis and treatment of clinical diseases, such as cancer [71], diabetes [72], and Crohn's disease [73].

To shed light on the brain-gut regulatory mechanisms, the relationship among clinical efficacy, gut microbiota, and functional connectivity of the brain network should be investigated. Via assessing brain connectivity with the structural and functional network analysis, it can be quantified that the functional and anatomical integrity and information flow in the whole brain network [74]. Thus, we will use a tripartite network analysis based on graph theory to inquire and visualize the relationship between clinical efficacy with gut microbiota and brain network connectivity.

Currently, positive control is one of the most commonly used control methods in randomized controlled trials (RCTs). The calcium channel blocker flunarizine, recognized as a first-line treatment for migraine [22–25], is widely used as a comparator in RCTs [37, 75, 76]. This control design is a good way to compare the efficacy, tolerability, and safety of different medical interventions for migraine. The present study is the first one to investigate the brain-gut regulatory mechanisms underlying different interventions (acupuncture versus flunarizine) for migraine.

Therefore, taking the brain-gut regulatory mechanisms as a starting point for this study, we will combine brain neuroimaging and gut microbiological data to analyze the similarities and differences of acupuncture and flunarizine treatment for migraine. This will permit investigation of the correlations between clinical efficacy and changes in gut microbiota and brain network function elicited by acupuncture and flunarizine treatment based on tripartite network analysis. It is hoped that the findings will inform efforts to partially reveal the common and different mechanisms of acupuncture and flunarizine on the treatment of migraine.

## Figures and Tables

**Table 1:** Data measurements at each time point.

Measurements	Enrollment	Allocation	Postallocation				Closeout
	−4 weeks	0 week	1 week	2 weeks	3 weeks	4 weeks	16 weeks
Eligibility screen	×						
Informed consent	×						
Physical examination	×						
Allocation		×					
Headache diary	×	×	×	×	×	×	×
Visual analog scale		×				×	×
Gastrointestinal symptom rating scale		×				×	×
Beck anxiety inventory		×				×	×
Beck depression inventory		×				×	×
Migraine-specific quality of life questionnaire		×				×	×
Magnetic resonance imaging		×				×	×
Gut microbiota examination		×				×	×
Adverse events			×	×	×	×	×

**Table 2:** Details of acupuncture treatment used in this study.

Classification	Headache attack site	Acupoints	Location
Main prescription		Fengchi (GB20)	At the level of Fengfu, in the depression between the upper ends of the sternocleidomastoid trapezius muscles
		Shuaigu (GB8)	Directly above Jiaosun, 1.5 cun above the hairline
		Baihui (DU20)	On the middle of the head, 7 cun directly above the midpoint of the posterior hairline

Shaoyang headache	Headache only attacks temporal side	Waiguan (SJ5)	On the line joining Yangchi and the tip of elbow, 2 cun above the dorsocarpal transverse crease, between the ulna and radius
		Yanglingquan (GB34)	In the depression anteroinferior to the head of the fibula

Taiyang headache	Headache involving the occiput	Kunlun (BL60)	In the depression between the lateral malleolus and the Achilles tendon
		Houxi (SI3)	Slightly making a fist, at the posterior end of the distal palmar crease of the 5 metacarpophalangeal joint and at the dorsoventral boundary

Yangming headache	Headache involving the forehead	Hegu (LI4)	Between the 1^st^ and 2^nd^ metacarpal bones and in the midpoint of the radial side of the 2^nd^ metacarpal bone
		Neiting (ST44)	In the anterior depression of the 2^nd^ and 3^rd^ metatarsophalangeal joints of the dorsal foot

Jueyin headache	Headache involving the vertex	Taichong (LR3)	In the depression anterior to the junction of 1^st^ and 2^nd^ metatarsal bones
		Neiguan (GB40)	On the line joining Daling and Quze, between the tendons of palmaris longus and flexor carpi radialis, 2 cun above the transverse crease of the wrist

## Data Availability

The submitted manuscript is a study protocol which includes no primary data now. Further information unaddressed can be obtained on reasonable request from the corresponding author in the manuscript.

## Abbreviations

MwoA: Migraine without aura
SPIRIT: Standard Protocol Items: Recommendations for Intervention Trials
ChiCTR: Chinese Clinical Trial Registry
TCM: Traditional Chinese Medicine
MRI: Magnetic resonance imaging
GSRS: Gastrointestinal symptom rating scale
BAI: Beck anxiety inventory
BDI: Beck depression inventory
MSQ: Migraine-specific quality of life questionnaire
TR: Repetition time
TE: Echo time
FOV: Field of view
SPSS: Statistical product service solutions
CONSORT: Consolidated standards of reporting trials
STRICTA: Standards for reporting interventions in clinical trials of acupuncture
CRFs: Case report forms
rRNA: Ribosomal RNA
OTU: Operational taxonomic unit
FSL: FMRIB Software Library
RCTs: Randomized controlled trials.
